# Reviews and Book Notices

**Published:** 1879-12

**Authors:** 


### Reviews and Book Notices.

---

ARTICLE XII.—CONSPECTUS OF ORGANIC MATERIA MEDICA AND PHARMACAL BOTANY. By L. E. Sayre, PH.G.

This work reminds one of the statement of Prof. Huxley, that if he had power he should cut out ruthlessly botany and materia medica from the course in a medical college. The book contains just that portion of materia medica which Prof. Huxley declared he would cut out, viz., "a knowledge of drugs and what they come from, and the animals and plants that yield them." Certainly, when we consider the short period devoted to medical education, and the vast number of facts which need to be learned about the physiological and therapeutical action of remedies, there is no time for the medical student to spend upon botany or upon the physical properties of drugs, except so far as they have some application to the art of healing. For the pharmacist, the book has undoubtedly a useful place to fill. J. H. S.

ARTICLE XIII.—THERAPEUTICS, MATERIA MEDICA AND TOXICOLOGY. H. C. Wood. Third edition.

This work has obtained an acknowledged place as the most complete work on the physiological action of remedies, in the English language. The value of the work to the practical student would be much increased by adding to the discussion of the physiological action of every remedy a summary similar to that which follows the account of the action of digitalis on the heart. The third edition contains some new articles, and other articles have been re-written to keep pace with the progress of science. The size of the volume is increased by about fifty pages.

J. H. S.

ARTICLE XIV.—FIRST STEP IN CHEMICAL PRINCIPLES. By Henry Leffmann, M.D., Lecturer on Toxicology in the Summer School of Jefferson Medical College.

In a small book of fifty-one pages, the author has given a succinct statement and careful explanation of the principles of chemical nomenclature, notation and the working out of reactions. To understand and remember a chemical formula, and to write a chemical reaction, are frequently very difficult for beginners in chemistry, and the ingenuity of the teacher is often severely tasked to explain the principles involved. This book will doubtless prove a help to both teacher and pupil. J. H. S.

ARTICLE XV.—CHEMISTRY, GENERAL, MEDICAL AND PHARMACEUTICAL. Attfield. Eighth edition.

This work is an extremely useful book of reference for the practitioner, containing the whole of the chemistry of the pharmacopeias of the United States, Great Britain and India, and is, at the same time, the best laboratory guide for medical students. The eighth edition contains new matter, which is represented in the index by three hundred new references, and increases the size of the work by twenty-three pages. J. H. S.

ARTICLE XVI.—GUIDE TO THE EXAMINATION OF URINE, With Special Reference to the Diseases of the Urinary Organs. By K. B. Hoffman, Professor at the University of Gray, and R. Ultzmann, Docent at the University of Vienna. From the second edition. Translated and edited by F. Forcheimer, M.D., Professor of Medical Chemistry at the Medical College of Ohio, Cincinnati.

The ideal of a work on urinalysis, for the practical physician, has not yet been reached. We have several exhaustive works, but what the physician needs is a work which shall give him a clear exposition of the methods which he can use in daily practice. Especially is this the case with quantitative determinations. Very few physicians have the time or skill to use gravimetric or even exact volumetric methods of analysis. At the same time, it is possible for them, and also convenient, to apply certain approximative methods which, although not strictly accurate, are sufficiently so, for application to chemical investigations. These methods are almost entirely omitted by the work before us, while exact gravimetric and volumetric processes are given. This does not justify the statement in the preface that "every test, every method, is brought home to the student and physician for use in practice." The work seems to be well translated, and is a very readable book. The addition of a few simple tests, to supply the deficiencies above mentioned, would make it an excellent work. J. H. S.

Article XVII.—Hygiene and Public Health. Edited by Albert H. Buck, M.D. New York: William Wood & Co. Two volumes. Octavo.

These two volumes form a work which will be welcomed by a large and increasing class of men. To the pin-feathered sanitarian, picking up his first crumb at the public crib, it will prove a friend in need — a friend indeed. By the student of nature, who delights in patient search for the ultimate causes of phenomena, it will be cordially received as a contribution towards the advancement of science. It will be esteemed by the philosopher as a mile-stone on the pathway of progress. Whatever may be said of its contents, it cannot be denied that it is an epoch-making book and its influence will be far-reaching and long continued. Fortunately, therefore, it is a work which in the main deserves commendation. Formed, as it is, by a series of unconnected essays, which have been prepared by different authors, treating of a great variety of subjects, there is, necessarily, great inequality of value in the different sections of the book. Certain topics seem to have been too lightly touched, while others have been elaborated out of proportion. Still, every part is instructive, if incomplete; and suggestive, if inaccurate.

The introduction forms an array of sixty-three pages, by the well-known Dr. J. S. Billings. It is a very readable paper, quite free from the extravagances which characterize those sanitary enthusiasts whose effusions provoke the inextinguishable laughter of gods and men. In fact, the doctor does good service in calling attention to the errors of judgment and of prevision into which such people continually fall, to the great detriment of the good name of sanitary science. He clearly shows the unwisdom of expecting too much from public sanitation and points out with great clearness the difficulties—pecuniary and the like—with which communities have to contend when they undertake the work of sanitary improvement. He specifies, also, the fact — too often overlooked — that the health and longevity of the members of a given community cannot be greatly affected by improvement of its sanitary condition. It is true that the introduction of pure water or the construction of extensive drainage works will, at first, in unhealthy locations, produce a very considerable reduction of the local death-rate. But this is only temporary. In a short time new causes become operative to a degree that compensates for the removal of the old; and something near the former rate becomes established. If, by vaccination, you diminish the mortality from small-pox, you soon have an increase from scrofulous diseases. If the mortality from infectious fevers decreases, the deaths caused by violence, by accident, by intemperance and vice increase. Suppress disease; old age and starvation will soon fill up the contingent of death. This necessary consequence of the co-relation of forces is too much ignored by our sanitary friends. It has not escaped the notice of statisticians who are concerned with life insurance. They declare that, during the past forty years, in which sanitary science has figured so largely in England, the death-rate has remained unchanged. This, however, need not discourage our efforts in behalf of improvement, for, if we may not hope for a longer average duration of life, we may at least hope to live more agreeably. The majority of young women would prefer to die of consumption rather than to live adorned with the scars of small-pox; and even if an epidemic of cholera does not enlarge the aggregate of mortality, pure water is a comfort that may not be despised. It is, certainly, more respectable to die of pneumonia than of yellow fever. Quite unnecessary, therefore, is the effort which writers are recently putting forth, in view of the above-mentioned statistical fact, to show that even a stationary death-rate is owing to sanitary measures and is, therefore, a positive gain to the cause of public health. This sounds wonderfully like the common professional plea, that, although the patient had not recovered his health, he certainly would have died if it had not been for the doctor's medicine. The fact is simply this: an equilibrium between the forces that maintain life and the forces which tend to death has, during the last forty years, been established and maintained in England. Such an equilibrium tendency is a law of nature everywhere and it is impossible for the human will to effect any great or permanent alteration of that equilibrium. A new sanitary superintendent, a general civic cleansing, an improved water supply, the cessation of a world epidemic, may affect the equation for a brief period, just as a conjunction of the outer planets may, for a few months, deflect the pathway of the earth around the sun; but the general course and result of forces is, in the long run, the same. And, as in the universe there are vast and varied forces, which produce slow and cyclical changes among the heavenly bodies, such as are implied by the changes which have taken place upon the earth during the lapse of geological time, so the public health is subject to vicissitudes of equilibrium which require centuries for their evolution and which are quite beyond the control of human will. For example, a review of the social history of the race shows that in Europe, for many generations, long before hygiene was ever thought of, the public health has been rising, the average duration of life has been growing, even in communities where social conditions and habits had undergone no change other than the natural outcome of increasing intelligence, morality and civilization. These are the great agents of improvement and to them must be ascribed the amelioration of the condition of the masses in Europe which has taken place during the past three hundred years. Stringent sanitary legislation, "shot-gun" quarantine, placards on houses, official inspection and supervision, in short, all the horrors of barbarous despotism, were utterly unavailing against the leprosies and pestilences of antiquity. It was the sun of civilization, opening to view the waste places of the earth and revealing space and liberty, stores of food and unheard-of comfort for the half-starved serfs of mediæval Europe, that banished leprosy and the plague from that quarter of the earth. It was the entrance of mankind upon that ample career which was opened by the discovery of America that accomplished results which the combined authority of law and religion could never effect. If history teaches anything, it teaches the lesson that public health depends not upon the existence of a learned and privileged class who shall govern men in such a way as to make them healthy. That method has been sufficiently tried and has failed. Public health depends rather upon the universal diffusion of knowledge and of wealth sufficient to put that knowledge into practice. As a community grows in wealth and intelligence, that is, as it becomes emancipated and free from the dominion of even a benevolent despotism, so will rise its standard of health and comfort. The highest aim, therefore, of the philanthropic sanitarian should be the diffusion of knowledge and the general distribution of property. Everything else will follow as a matter of course. By this method, only, can success be attained; but it is exceedingly difficult to get ordinary men to trust in the operation of the natural laws of the universe. Even a thinker ordinarily as clear-headed as Dr. Billings cannot always rise above the official bias due to his position, as appears from his utterance regarding small-pox (p. 8.) to the effect "that it should never appear on the death register; yet, to obtain such an universal and satisfactory vaccination and revaccination of each individual as will give this security, there is necessary the decided and persistent interference of government to an extent which has not yet been provided in this country except in a few limited localities." Now in England and on the continent of Europe, the government does interfere to secure the vaccination of everybody; but with what success, the history of the frequent local epidemics of small-pox abundantly shows. Our own experience in this country, where compulsory vaccination is not practiced is at least as favorable as the result of paternal government. And in countries where the compulsory method is in vogue, there is always an angry resistance manifesting itself against the law, which maintains the prejudices of the ignorant against the practice. Witness the English anti-vaccination leagues and literature. The best safeguard against the spread of small-pox is an occasional outbreak of the disease. Compulsory legislation can accomplish nothing better.

The official bias of the author again shows itself, in the very cursory manner with which he dismisses the matter of personal hygiene — (pp. 11, 12). This, by far the most important factor in the work of advancing the public health, seems to have very few attractions for the professional sanitarian, probably because it must be exercised in a sphere beyond the reach of public officials. It is, however, the agent which has effected the greatest part of the physical improvement of the human race; and to the diffusion of the knowledge upon which it depends must we look for all real progress in the future. Such diffusion will accomplish all, and more than all, the good results that might be expected from working "in accordance with the laws of natural selection, [to] prevent the reproduction of weak and unhealthy persons." It is indeed true that the immense results which are possible in this department of effort "must be effected by parents and teachers; by guiding the children under their charge into proper mental and physical habits."

The second chapter of the introduction is devoted to the "Causes of Disease." These the author divides into four classes: 1st, hereditary: 2d, physical and chemical; 3d, organized or vital; 4th, mental or emotional. Of these the first is considered as belonging chiefly to the unavoidable causes of disease. The second class is left for discussion in the special sections of the work. The fourth class is considered too large and difficult for discussion in a manual like the present. "The third class of cases (p. 13), viz., those which are known or supposed to be organized or vital in character, is the most important of all in public hygiene, and hence we shall dwell upon it a little." About twenty pages, accordingly, are devoted to a brief, but lucid summary of recent literature on this subject. The author adopts the German classification into:

A. Parasitic diseases.

B. Contagium diseases.

C. Miasmatico-contagium diseases.

D. Miasmatic diseases.

That certain diseases, such as scabies and trichinosis, are purely parasitic is beyond a doubt. That certain diseases may be propagated by the transfer of material particles from the body of a diseased person to the body of a healthy individual is also beyond a doubt. That certain diseases are produced by a similar exchange between certain soils and living bodies will not be qnestioned. The only debate arises over the nature of such transferable particles. Are they organized, living germs, or are they particles of non-living, decomposing organic matter ? That is the question. Dr. Billings criticises very freely the extravagancies and the confusion of ideas which are the principal characteristics of the advocates of the so called "germ-theory." He also very wisely condemns the notion which some have advanced, to the effect that it is a matter of no consequence, so far as sanitary administration is concerned, whether the germ-theory be right or wrong. It is a matter of great importance, for if the germ-theory, as ordinarily preached, is incorrect, then much that is now considered of prime importance in sanitary administration will sink into insignificance in comparison with personal hygiene.

As just now remarked, there is no question as to the dependence of certain diseases upon the invasion of the tissues by certain parasites. But regarding certain other diseases there is still an active controversy. Are diphtheria, splenic fever, relapsing fever, hog-plague and the like diseases to be ranked as idiopathic, endogenetic diseases, or are they to be always charged to the entry of infective spores into the blood ? Dr. Billings inclines toward the latter view of the subject, evidently almost persuaded by the clamor of Pasteur and the microscopists of his school; but the doctrine of these observers does not account for the existence of cases of splenic fever without the presence of bacteria, as reported by P. Bert, Joffroy, Hayem, Trosbot and others. Pasteur, it is true, denies the name of splenic fever to such cases, calling them cases of septicæmia; but his evasions and his failures to demon strate in puerperal fever the parasite which he had announced as the infective agent, along with his unsatisfactory notions regarding the relation of microphytes to the processes of fermentation and putrefaction, render one very slow to accept as an established fact the parasitic theory of these diseases. The same thing may be said of the experiments by cultivation and inoculation as practiced by Koch and by Klein. They cultivated successive generations of poisonous bacteria and succeeded in producing splenic fever by inocculating animals with nine or ten consecutive crops of the parasite. This certainly indicates a power on the part of the bacteria, to produce a substance poisonous to the inoculated animal, but it does not prove that the original source of the poison resided in bacterial germs. It is acknowledged that the harmless bacteria subtilis and the poisonous bacteria anthracis are, for all practical purposes, morphologically identical. Now the real question is this: Are bacteria anthracis anything more than the ordinary bacteria subtilis undergoing a process of disease, resulting from immersion in the liquids of a specifically diseased animal. We know that the form, and modes of growth of parasitic microphytes may be varied almost beyond description by alterations of the fluids in which they are developed. If now, a family of bacteria are infected by the disease of their host, it would not be at all singular if they should transmit a hereditary disease to their offspring during the process of "cultivation." But, inasmuch as hereditary diseases tend to self elimination, it is necessary, in order to decide the question at issue, to pursue the method of cultivation and inoculation with successive generations to far greater length than has yet been done. Nine or ten generations might very well be insufficient to give opportunity for reversion to a healthy ancestral type, when, perhaps, a hundred successions might exhibit the process in the clearest manner. Until some such crucial test shall have been applied, we must side with Billroth in the opinion that these microphytes may be the carriers, but not the original sources of the infective virus. In fact, it is only by continually keeping in mind the broad generalizations of Liebig as expressed in his doctrine of fermentation, that it is possible to reconcile the conflicting hypotheses of different writers, and to clear the doctrine of infection of all the rubbish that has been heaped about it by one-eyed microscopists. Great confusion has arisen out of the failure to distinguish sharply between organized bodies and their products. A very large proportion of the advocates of the germ theory seem to make no distinction between the properties of organized matter and of organic matter—between a living cell and its excretion; consequently we find such writers making no distinction between the activities of these very different entities. Because a cell may multiply itself by fission or otherwise, they seem to think that the waste products thrown off by a cell may multiply themselves indefinitely in a similar manner; but we know how cells multiply themselves, and we know something of the way in which defected, non-living organic matter behaves itself. The numerous ferments which are elaborated by specialized cells of the body, though non-living, are exceedingly potent—even after all connection with the generative cell has long ceased—to effect change in protoplasmic matter, both outside of the cell, as in the process of peptic digestion, or inside of the cell, as when splenic ferment reaches and modifies the function of pancreatic cells. In all such cases the active ferment operates by exchange of molecular motion, and the ferment is reproduced, not by any generative process, but by the continued life and modification of cells whose activity is sustained by the remote consequences of the action of the ferments which they have produced. The same process goes on with new forms of intensity in certain diseases. The cells of a variolous patient produced small pox virus. That virus invades the blood of a healthy subject; it modifies the nutrition of every cell in his body until they too yield a virus which exactly resembles the first. A cow becomes plethoric, and develops splenic fever. The morbid cells of her tissues excrete a poisonous virus which pervades all the liquids of the body, and even infects the bacteria which soon swarm among the non-resistent tissues. Microphyte and microzoon (animal cell) then alike produce the poison and discharge it into the blood and lymphs of the animal until everything connected with the victim is alike infectious. In a similar manner the contagium of scarlet fever and of kindred diseases may be supposed to originate and to be multiplied. In cholera and yellow-fever it is possible that these non-living cell-excreta may need a farther change outside of the body before their full power for evil can be developed. Viewing the subject in this way, we can reconcile the doctrines of Liebig and of Pasteur, and can obtain a much clearer notion of the manner in which infective diseases may originate and are propagated, than if we adopt the so called "germ theory" with all its visionary outfit of invisible spores and parasites too small for sight, yet large enough to drain a fever-patient of his fluids till he must cry continually for water to make good the loss!

The necessary limits of a review like the present render it impossible to elucidate this subject. We can only throw out these few hints which may suggest thought to the initiated, but which, of course, can convey no intelligible idea to those who are destitute of scientific training. The whole search for "germs," as conducted by the majority of the microscopists who are continually cackling over new found forms of morbid growth in the blood of all manner of patients, affords a striking illustration of the ease with which one may fail to discover the simplest thing for which he has not been trained to look. In their eagerness after bacteria they have quite neglected the cells of the body which in the vast majority of diseases, if not in every instance, are the seat of the essential morbid processes, and the sources of infective matters which are discharged into the liquids of the body. This being the fact, a little thought will enable one to see how much more important are measures which tend to sustain the vitality and the normal function of the cells of the tissues than any simple attempt to keep bacteria away from the person. In other words, the personal hygiene and therapeutics, which some of our enthusiasts are so disposed to undervalue, are of far greater importance than any process of quarantine against "germs." Dr. Ezra M. Hunt is almost the only recent American sanitary author who seems disposed to give rightful prominence to this branch of hygiene; and yet it is the principle which underlies such measures as vaccination and all our rational therapeutics. Once possessed by a comprehension of this great truth, it will no longer be living matter, but dead, decomposing matter, which we shall seek to avoid. We shall think less of antiseptics and more of abundant oxygen.

The third section of the introduction treats of the "Jurisprudence of Hygiene." This is, perhaps, in some respects, the least satisfactory portion of the paper, for the very sufficient reason that sanitary legislation is yet in its infancy; and, having been, so far, for the most part, dictated by half-educated enthusiasts, and interpreted by lawyers and judges who are more or less under their influence, there is yet no complete and final adjustment of the subject upon a basis of true science and sound philosophy. One of the very first propositions with which our author starts, viz., that it is necessary in this generation to establish public hygiene upon the foundation of utilitarianism (p. 35), is a very good example of the narrow philosophy which guides our sanitarians. *Expediency* and *utility* may be allowed to suggest motives to the common herd, but the highly-gifted few — the benevolent despots whom we are advised to set up over ourselves — must act upon a higher level than is furnished by these narrow motives, if they wish to reconcile us to their dominion. They must ask what is in conformity with the known laws of the universe — what is *right* and what is *wrong* — if they wish to secure a wide and lasting influence with the public. This is where so much of our sanitary administration is weak. It busies itself too much with the utilities; and utility, for the average sanitary official, too often relates merely to the bearing of particular measures upon his chances for keeping his hand in the public treasury. But this is not all. As President Woolsey (p. 36) and Herbert Spencer, and numerous other writers upon the philosophy of government, have clearly shown, the whole matter of sanitary legislation for the benefit of the public health is muddled by the attempt to accomplish with the same machinery of government and taxation two things which cannot thus be confused without injustice. As Bastiat says: "The government cannot organize labor and education and public health and charity and such things without disorganizing justice." It is the duty of the government to provide uniform protection for the lives, liberties and property of all its citizens, and consequently it is the duty of the government to tax its subjects with perfect uniformity. Each citizen should pay the same amount for the police protection of his life and liberty that every other citizen pays. For the protection of his property each one should pay according to the value of that property. If it costs ten dollars to protect the lives of A and B, then A and B should pay five dollars each. If it costs one hundred dollars to protect their houses and other property, and if A owns nine-tenths of that property, then his just proportion of the total tax for person and for property is ninety-five dollars, while B should contribute but fifteen. That is the just way to raise money for the support of government. But when it is proposed to tax people for the protection of their health, it becomes impossible to adjust such a tax with anything like uniformity, and it becomes an unjust tax, because bearing with the utmost inequality upon individual citizens. Because Mr. Smith is rich, and chooses to use his income in such a way as to preserve the health of himself and his family, it is no reason why he should be taxed to also pay for the improvement and supervision of the families of poor Harry and drunken Dick. Benevolence is no legitimate part of the function of civil government. All governments undertake to dispense more or less of compulsory charity — a hereditary custom derived from the despotic ancestors of existing governments — but it is a practice fraught with evils, and is the parent of dangers to which we in this favored land have not yet opened our eyes. Benevolence is the duty of private individuals; protection of life, liberty and property is the only proper function of government. Consequently, the care of the poor, the ministration to the sick, and the elevation of the downcast, should be left to private enterprise. Whenever it can be shown that a particular measure interests the welfare of all alike, it will then be proper to levy the cost of the undertaking upon all. Such a measure is the supply of pure water to every inhabitant of a city, where the natural sources are deleterious. Such a measure is the maintenance of quarantine against those foreign pestilences which can be kept out by such expedients. Such a measure is the cleaning of the streets and the privies, which all must use. But the attempt to protect citizens at the common expense against their local endemic diseases does not belong in this category, for the reasons previously given. That is the field for personal, individual responsibility, effort and expense. This is the position taken by the highest philosophy, and Dr. Billings would have done better for his own reputation if he had sided with Woolsey and Spencer. Had he done so, he would not have fallen into the inconsistency which he manifests, in common with the majority of sanitarians, when he argues (p. 35) that because health is part of a man's capital, it must be protected by the government, for the reason that the State undertakes to protect all property. We will admit that health is a valuable possession, and that it should be protected just like all other property. Now the way a just government treats property is not by managing it for the benefit of its owners, but by providing equal security to all in the possession and use of their valuables. That done, every man is free to use his property as he pleases. He may give it away, or destroy it, or increase it, and no man shall interfere. In like manner the health of every man is his own property, and it is a possession of equal value to every man. Consequently, no government has any right to dictate, as our sanitarians desire, how a man shall use or abuse his health, nor has it any right to tax one man more than another for the protection of the collective health of the community.

It really seems almost a waste of time to dwell with such minuteness upon these elementary propositions ; but the fact that a writer of such ability as Dr. Billings has failed to perceive their bearing, shows how much our sanitarians have to learn from philosophical thinkers and teachers, even though they be as obscure and unknown as President Woolsey, or Herbert Spencer, or John Stuart Mill. However, the public does not propose to be coerced by philosophers or by sanitary experts ; consequently it is useless for either class to attempt the forcible realization of their opinions in practice. The only thing that can properly be done is to insist upon a conscientious presentation of the highest truths in science and in philosophy by those who devote their lives to research ; for we may be confident that the advanced science and philosophy of to-day will supply the common creed of to-morrow. Every attempt to interfere with the orderly evolution of nature in these respects will only hinder progress, and will recoil injuriously upon its authors.

Passing to a consideration of the legal relations of physicians to the public in the matter of statistical returns, and registration of diseases, it is pleasing to find our author ranging himself on the side of justice in his affirmation of the obligation of the State to pay for all such service. The experience of New Hampshire, Connecticut and Minnesota, and of British cities which have undertaken a registration of infectious diseases, does not, however, reveal any practical difficulties in the way of fairly compen sating physicians and others for their assistance in the matter. The only difficulty that can interfere grows out of the immoral unwillingness of so many people to pay for work which they desire to have done. In this disposition, it is to be regretted, that communities are too often encouraged by political doctors who hope to magnify their own importance by the unremunerated services of their non-official brethren, and by certain enthusiasts, bitten with a mania for statistics, for the sake of which they are willing to sacrifice honor, justice and everything else. In this connection it is noteworthy that the official bias of our author has in some degree overcome his usually candid disposition, a fact which is shown by his absurd notions (p. 46) that the compensation of physicians for services to the State "should be indirect, in the shape of certain privileges," such as "a voice in the selection of the health official or officials with whom they are to co-operate, or, some permanent and organized means of representation in the councils of the sanitary authority." In reply to this it is sufficient to remark that in this State, at least, the organic law expressly forbids the creation of any privileged class whatever. As for voice in the selection of the health officials, that is too unsubstantial a privilege to excite anything but laughter among the initiated, and as for representation in the sanitary councils, that is too empty and impracticable a consideration to be esteemed by any but the official classes. The vast majority of physicians are plain men who practice medicine, not for the sake of science, or for political influence, but for the sake of the shekels which they may thus honestly earn, and nothing else has any tangible value as a compensation for their labor. No lawyer would ever think of giving his services as a notary public for the privilege which he already possesses of voting at the primaries, or for the chances of some day becoming a judge. A voice in the selection of health officials and a representation in sanitary councils is already the property of the medical profession, and its value cannot be increased by any mere legal formula.

The concluding portion of the paper treats chiefly of the proper functions of the boards of health. The author admits that the legislation necessary for their establishment and guidance presents great difficulties. There is danger, he thinks, of conferring too much power by vague legislation and there is danger of too much prolixity and rigidity if the health laws are made specific in their provisions. These difficulties are greatly over-rated by sanitary writers. They grow out of ignorance of the legitimate functions and limitations of government. The practical difficulties are largely caused by the hereditary inclination of legislators and public officials to intrude their supervision and authority into the private life of citizens. It was once thought right for the state to govern the consciences of men in all matters pertaining to religion. This primitive notion we have nearly outgrown; but the analogous superstition that the state must take charge of the health of its citizens, is exceedingly fashionable and will always be so while money and political position can be made by such work. When our legislators become willing to listen to the teaching of those philosophers who, like Herbert Spencer believe in a moral order as the basis of civil government, we shall not find it so difficult to secure sanitary legislation which will be simple and comprehensive alike. In this connection it is encouraging to note the action of the supreme court of Massachusetts, by which the right of trial by jury has been restored to the citizens of that State in cases of appeal from the decisions of the State board of health. The authority which had been thoughtlessly conferred virtually upon the secretary of the board, making him the supreme arbiter of the most important interests, was an act so completely opposed to the fundamental principles of free government that its abrogation by the supreme court should really be a matter of national thanksgiving.

The recent legislation, establishing a State board of health in Illinois and the national board of health in Washington, is duly noticed by the author. Our own State is credited with the possession of the most potent board in the country so far as its theoretical powers and privileges are concerned. As for their practical working, Dr. Billings shrewdly and correctly remarks that "among observant sanitarians there is a fear that if matters take the usual course, there will be a reaction and that the description of the course of public hygiene in England—viz., that it has consisted in taking three steps forward and two backward, will also apply here" (p. 57). Mere sanitarians may "fear" such a consummation but it will have no terrors for the thoroughly scientific observer, for such is the universal law of all rational human progress. Enthusiasts rush wildly in advance, throwing out a disorderly skirmish line, behind which the cool-headed generals select an impregnable position to which all must retire when night puts an end to the combat.

With these remarks we must leave this very able paper, which, though perhaps not in all respects fully up to the high water mark of science and philosophy, will, if read with discrimination do a great deal of good.

After the introduction, the first paper is devoted to the subject of "Infant Hygiene." It is sufficient praise of this essay to note the fact that it is the work of Prof. A. Jacobi, the well-known writer and teacher and repository of all that is known about the care of children.

"Food and Drink," is the title of the next paper. It is written by Prof. James Tyson, of the University of Pennsylvania, and it forms an excellent summary of the teachings of physiology in this department.

Prof. Wm. R. Nichols, of Boston, Mass., writes on "Drinking Water and Public Water Supplies." This forms a valuable exhibition of what is known concerning the different kinds and sources of water that are used in different parts of the world. The subject of impurities and means of purification is very sensibly treated. The presence of microscopical forms of plant and animal life is not considered especially objectionable. The deleterious effects of sewage and other artificial causes of pollution seem to depend upon the degree of concentration rather than upon the quality of the impurity. As for the presence of specific "germs" of disease in water, the professor does not adopt any particular theory. He simply and correctly states that the weight of opinion inclines to the view that decomposing nitrogenous matter of organic origin is the most dangerous form of injurious matter. This, when derived directly from diseased bodies is, of course, the most concentrated and most virulent. When it has infected algous or fungous growths, they may also become capable of discharging virulent matters like those by which they were originally diseased. In this way such minute organisms may become associated with the propagation of diseases dependent upon decomposition of specific organic matters which they did not primarily originate.

A paper on "Physical Exercise," is contributed by A. Brayton Ball, M.D., of New York. This is also a concise and useful summary of the physiology and pathology of muscular motion.

Arthur Van Harlingen, M.D., an eminent dermatologist, in Philadelphia, contributes a brief essay on "The Care of the Person." All very good. He advocates the establishment of public baths in our cities — a very desirable thing; but he neglects to remark that such luxuries should be provided by charitably-disposed citizens rather than by public taxation.

The great subject of "Soil and Water" is treated at length by Wm. H. Ford, M.D., of Philadelphia. The whole matter of sewerage is fully considered. Contamination of the soil by privies, and the remedies, are also passed in review. The author writes very sensibly of the relation which exists between certain diseases and certain conditions of the soil, air and water. The upshot of the immense number of observations and opinions which he has collected, but only partially analyzed, is not at all favorable to the so-called "germ theory." Nor can it be otherwise, for the only living germs concerned are the cells of the body. When inundated soils containing protoplasmic matter — whether of animal or vegetable origin is of secondary consequence — are exposed to heat and oxygen, the dead protoplasm decomposes. This detritus is dissolved in water; or, in a state of minute subdivision, may be transported by the air. By either vehicle it may find entrance into the liquids of the body. Bath ing the cells of the body, these contaminated fluids will seriously modify the nutrition of those cells. If the quantity of impurity be not too great, the vital forces of the cells soon adjust themselves to the novel disturbance; but if the concentration of the poison exceed certain limits, the cells may pass into a specific condition of disease, or may succumb to new and extraordinary concurrences of external force. Thus viewing the subject, it is easy to see how "telluric emanations" in a temperate climate, where animal and vegetable refuse mingle in the soil, may produce typhoid fever *de novo;* how vegetable detritus alone, under analogous conditions, may produce malarial fever; how the concentrated animal and vegetable filth of a tropical city, festering in the long-continued heat of a southern summer, may produce yellow fever, etc., etc. Thus produced, the propagation of those diseases, like typhoid and typhus, which are dependent chiefly upon animal poison, becomes very easy. In typhus, the infection may, perhaps, be derived directly from the body of the patient. In yellow fever and in cholera the detritus of diseased cells seems to need farther decomposition or combination outside of the body before it can become infective. This is really the only germ theory that has any logical basis in facts. But the limits of this review will not permit further discussion of the subject.

Dr. D. F. Lincoln, of Boston, Mass., contributes the next paper, an exhaustive review of all that is known about "The Atmosphere." This is considered under four heads: I. Normal Components of Air. II. Impurities, Organic and Inorganic. III. Meteorology and Climate. IV. Ventilation and Heating. The essay treats of all these topics with encyclopædic fullness, forming a body of valuable information which will not admit of abstraction.

The first volume is concluded with an excellent essay on "Hospital Construction," by Francis H. Brown, M. D., of Boston, Mass.

(*To be continued.*)

